# EHMTI-0048. Epigenetic changes in a rat model of migraine with aura

**DOI:** 10.1186/1129-2377-15-S1-A6

**Published:** 2014-09-18

**Authors:** M Vila-Pueyo, N Fernández-Castillo, B Cormand, P Pozo-Rosich, A Macaya

**Affiliations:** 1 Paediatric Neurology Group, Vall Hebron Research Institute, Barcelona, Spain; 2 Genetics Department, University of Barcelona, Barcelona, Spain; 3 Headache and Neurological Pain Group, Vall Hebron Research Institute, Barcelona, Spain

## Introduction

Migraine with aura (MA) is a subtype of migraine characterized by reversible neurological disturbances preceding headache. Epigenetic mechanisms are postulated to mediate migraine susceptibility. MA neurophysiological correlate is cortical spreading depression (CSD), a wave of neuronal depolarization and depression that is suppressed by chronic administration of the migraine preventive drugs valproate and topiramate.

## Aims

Using a CSD rat model, we investigated if valproate and topiramate lower the susceptibility to develop CSD by inducing changes in brain DNA methylation.

## Methods

Adult male Sprague-Dawley rats were treated with valproate, topiramate or saline for 4 weeks. CSDs were KCl-induced for 1 hour. Cortices were removed and DNA was extracted to perform MBD-based genome-wide methylation sequencing. Results were analyzed by MEDIPS software to obtain differentially methylated regions (DMRs). Genes containing DMRs were analyzed for GO and KEGG pathways enrichment and gene networks were constructed using IPA software.

## Results

Both treatments, as expected, reduced CSD intensity. Both drugs induced differential methylation (DM) of genes belonging to “synapse activity” pathways including Rimbp2 and Grip1. Treatment with valproate was associated with DM of ion channel encoding-genes such as Asic2 and Kcnd2. CSD, in turn, promoted DM of genes involved in axon growth (Cdh13), modulation of pain (Mrgprx3) or neuronal proliferation (Mapk10).

## Conclusions

These results confirm that valproate and topiramate protect against CSD in the rat and that this is associated with changes in brain DNA methylation. Further analyses are required to determine the potential of the identified targets in determining susceptibility to human migraine.

No conflict of interest.

